# Effect of electrical stimulation for chemotherapy-induced nausea and vomiting in patients with liver cancer

**DOI:** 10.1097/MD.0000000000015255

**Published:** 2019-04-19

**Authors:** Wei-hong Li, Dong Li

**Affiliations:** a Department of Emergency Surgery, First Affiliated Hospital of Jiamusi University, Jiamusi; b Department of Hepatobiliary Surgery, The First People's Hospital of Xianyang City, Xianyang, China.

**Keywords:** chemotherapy-induced nausea and vomiting, effect, electrical stimulation, liver cancer

## Abstract

**Background:**

This study will be proposed for assessing the effects of electrical stimulation (ES) for chemotherapy-induced nausea and vomiting (CINV) in patients with liver cancer (LC).

**Methods:**

We will identify the relevant literatures of ES for CINV in patients with LC from following databases: Cochrane Library, PUBMED, EMBASE, Web of Science, the Cumulative Index to Nursing and Allied Health Literature, the Allied and Complementary Medicine Database, Chinese Biomedical Literature Database, and China National Knowledge Infrastructure from inception to the date of literature searched without any language restrictions. Randomized controlled trials and case–control studies on assessing of effects and safety of ES for CINV in patients with LC will be included. Methodological quality for all included studies will be assessed by using Cochrane risk of bias tool. RevMan 5.3 software (Cochrane Community, London, UK) will be used to analyze the data.

**Results:**

This study will summarize current evidence for ES on CINV in patients with LC. Primary outcome includes symptoms severity. Secondary outcomes consist of appetite, performance status, health-related quality of life, and adverse events.

**Conclusion:**

The results of this study will provide latest evidence to judge the effects and safety for ES on CINV in patients with LC.

**PROSPERO registration number:**

PROSPERO CRD42019126379.

## 1 Introduction

Liver cancer (LC) is the one of the most common cancers among the digestive system diseases.^[1–4]^ It has been reported that LC is the fourth most common malignancy cancer in China.^[5–8]^ It is also the third most common cancer-related deaths among Chinese population.^[5–9]^ Most importantly, it is difficult to cure because most patients are detected and diagnosed with LC until its advanced stage.^[5,6,9]^

Chemotherapy is recommended to treat LC and has achieved promising efficacy. However, when patients received this therapy, they often suffer from a variety of adverse events. Of those, chemotherapy-induced nausea and vomiting (CINV) is one of the most and worst common side effects.^[10–12]^ If this condition cannot be treated adequately and effectively, it may cause malnourishment and consequently impact the immune system, performance status, electrolyte imbalance, and quality of life of patients with LC.^[13–15]^ Most importantly, it may also result in treatment interruption or dose decrease, and then finally affect the LC cure rate.^[16,17]^

Antiemetic agents are utilized to treat CINV, such as 5-HT3 receptor antagonists, steroids, cannabinoids, and antihistamines.^[18–20]^ However, these treatments still have limited efficacy for some patients. In addition, they also have some other significant side effects for those patients.^[18–20]^ Thus, alternative managements for such condition are urgent. Electrical stimulation (ES) is a very potential candidate. Several previous studies have reported to use such therapy to treat CINV and have achieved encouraging effectiveness.^[21–29]^ However, no study systematically addressed its effects for LC patients with CINV. Thus, this study will systematically investigate the effects of ES for LC patients with CINV.

## 2 Methods

### 2.1 Ethics and dissemination

No ethic approval is required in this study, because it does not analyze individual patient data. The results of this study are expected to be published at a peer-reviewed journal.

### 2.2 PROSPERO registration

This protocol has been registered in PROSPERO with CRD42019126379. It reports according to the Preferred Reporting Items for Systematic Reviews and Meta-analyses (PRISMA) Protocols statement guidelines.^[30]^

### 2.3 Eligibility criteria for study selection

#### 2.3.1 Types of literature

This study will include randomized controlled trials and case–control studies that assess effects and safety of ES on CINV in patients with LC. However, any other studies, including nonclinical studies, case reports, case series, observational studies, and studies without control group will all be excluded.

#### 2.3.2 Types of patients

All patients with LC receiving or have received chemotherapy are clinically diagnosed, as CINV will be included in this study. However, the vomiting and nausea resulted from other diseases, such as stomach cancer, or any other disorders will not be considered.

#### 2.3.3 Type of interventions

In the experimental group, patients can receive any forms of ES interventions, such as neuromuscular ES, electroacupuncture, etc. However, studies will be excluded if they utilized ES as well as other therapies for the treatment of CINV.

In the control group, apart from ES, patients can receive any treatments for the treatment of CINV.

#### 2.3.4 Type of outcomes

##### 2.3.4.1 Primary outcome

Primary outcomes included symptoms severity, as measured by Multinational Association of Supportive Care in Cancer, or other scales.

##### 2.3.4.2 Secondary outcome

Secondary outcomes included the following:

(1) Appetite, as assessed by Anorexia scale using visual analogue score, or other scores;
(2) Performance status, as evaluated by using Zubrod Performance Status score, and other instruments;
(3) Health-related quality of life, as measured by 36-Item Short Form Health Survey or other tools;
(4) Adverse events, including any expected and unexpected adverse events or adverse reactions.

### 2.4 Strategy of literature searches

The following electronic databases will be retrieved from inception to the date of literature searched without any language restrictions. These databases include Cochrane Library, PUBMED, EMBASE, Web of Science, the Cumulative Index to Nursing and Allied Health Literature, the Allied and Complementary Medicine Database, Chinese Biomedical Literature Database, and China National Knowledge Infrastructure. In addition, we will also search conference abstracts, dissertations, and reference lists of relevant studies. Any randomized controlled trials or case–control studies on assessing the effects of ES for CINV in patients with LC will be considered for inclusion. We will build a detailed search strategy for Cochrane Library in Table 1, and also apply similar search strategies to the electronic databases.

**Table 1:**
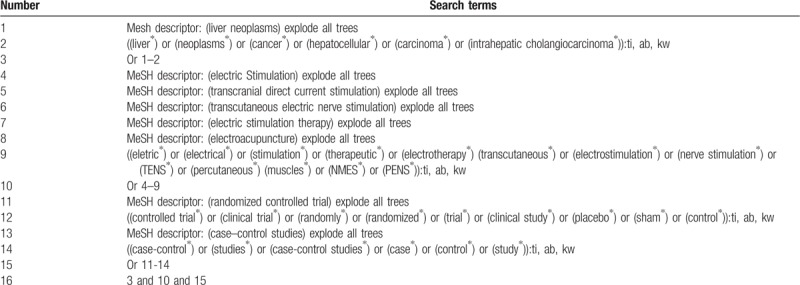
Detailed search strategy for database Cochrane Library.

### 2.5 Data collection

#### 2.5.1 Study selection

Two reviewers will independently and carefully scan the titles and abstracts for all records first, and all irrelevant studies will be excluded. Then, a further new round selection will be conducted by reading full papers at this stage. Any disagreements regarding the study selection between 2 reviewers will be consulted by a third reviewer. All processes of study selection will be presented in the PRISMA flowchart with detailed reasons of exclusion or inclusion for each study.

#### 2.5.2 Data extraction

All relevant information and data will be extracted by 2 independent reviewers according to the previous designed standard data collection sheet. It comprises of the following information: title, first author, year of publication, location, diagnostic criteria, eligibility criteria, patient characteristics, study methods, such as details of randomization, blinding, treatment details, and all outcome measurements. Any disagreements will be resolved by a third reviewer through discussion.

#### 2.5.3 Dealing with missing data

If there is any insufficient information or missing data during the process of data extraction, we will contact authors of original studies for the requirement. If we cannot inquire those data, only present available data will be analyzed.

### 2.6 Risk of bias assessment

Two reviewers will assess the risk of bias for all included studies independently. A third reviewer will be invited to help solve any disagreements arise between 2 reviewers through discussion. The Cochrane Collaboration Tool will be utilized to assess the risk of bias from seven perspectives, and each one will be further judged as low risk of bias, unclear risk of bias, or high risk of bias.

### 2.7 Statistical analysis

Statistical software RevMan 5.3 and State 12.0 (StataCorp, College Station, TX) will be implemented to pool the data and to conduct meta-analysis. For continuous data, mean difference or standardized mean difference and 95% confidence intervals (95% CIs) will be used to assess the extracted data. For dichotomous data, risk ratio and 95% CIs will be utilized to express the extracted data.

We will assess the heterogeneity among included studies with *I*^2^ test according to the Cochrane Handbook: *I*^2^ ≤ 50%, representing low heterogeneity; and *I*^2^ > 50%, representing high heterogeneity. If heterogeneity is low, a fixed-effect model will be utilized to pool the extracted data, and meta-analysis will be conducted. If heterogeneity is high, a random-effect model will be used to pool the data, and meta-analysis will be performed in accordance with the results of subgroup analysis. If heterogeneity is still high after the subgroup analysis, data will not be synthesized, and a narrative summary will be elaborated only.

### 2.8 Additional analysis

#### 2.8.1 Subgroup analysis

Subgroup analysis will be performed to investigate the potential source of high heterogeneity based on the different patient characteristics, treatments, controls, and outcome measurements.

#### 2.8.2 Sensitivity analysis

Sensitivity analysis will be carried out to check the robustness of the combined results by taking away the effect of missing data, and low methodological quality studies.

#### 2.8.3 Reporting bias

The reporting bias will be assessed by funnel plot^[31]^ and Egger regression^[32]^ when sufficient studies in this review (more than 10 studies) are included.

## 3 Discussion

CINV is a very common disorder for cancer patients receiving chemotherapy.^[10–12]^ Although several managements are recommended to treat this condition, their efficacy is not always satisfied, and also accompanied by additional adverse events, which greatly affect the cancer treatments.^[16,17]^ Thus, alternative therapies with fewer adverse events are still urgently needed for patients with CINV.

Several previous clinical trials have reported that ES is effective for LC patients with CINV, and can greatly relieve this condition.^[21–29]^ However, no study has systematically addressed this issue. Thus, in this study, we will systematically investigate the effects of ES for LC patients with CINV. It is very important to determine whether ES is an effective management for reliving CINV condition in patients with LC. The present study will help provide information to benefit either patients and clinicians, or the health policy-maker with a comprehensive understanding of the effects of ES management.

## Author contributions

**Conceptualization:** Wei-hong Li, Dong Li.

**Data curation:** Wei-hong Li, Dong Li.

**Formal analysis:** Wei-hong Li, Dong Li.

**Funding acquisition:** Dong Li.

**Investigation:** Dong Li.

**Methodology:** Wei-hong Li.

**Project administration:** Dong Li.

**Resources:** Wei-hong Li.

**Software:** Wei-hong Li.

**Supervision:** Dong Li.

**Validation:** Wei-hong Li, Dong Li.

**Visualization:** Wei-hong Li, Dong Li.

**Writing – original draft:** Wei-hong Li, Dong Li.

**Writing – review & editing:** Wei-hong Li, Dong Li.
